# Chemogenetic selective manipulation of nucleus accumbens medium spiny neurons bidirectionally controls alcohol intake in male and female rats

**DOI:** 10.1038/s41598-020-76183-2

**Published:** 2020-11-05

**Authors:** C. E. Strong, D. P. Hagarty, A. Brea Guerrero, K. J. Schoepfer, S. M. Cajuste, M. Kabbaj

**Affiliations:** Program in Neuroscience, Department of Biomedical Sciences, Florida State University, Medical Science Research, Room 3300-H, 1115 W. Call St., Tallahassee, FL 32306 USA

**Keywords:** Learning and memory, Molecular neuroscience, Motivation, Reward

## Abstract

The nucleus accumbens (NAc), considered the hub of reward circuitry, is comprised of two medium spiny neuron (MSN) subtypes that are classified by their enrichment of dopamine 1 (D1) or 2 (D2) receptors. While reports indicate that alcohol increases excitatory neurotransmission exclusively on NAc D1-MSNs in male rats, it remains unknown how NAc MSNs control alcohol intake in either sex. Therefore, this study investigated how NAc MSNs mediate alcohol intake by using Drd1a-iCre and Drd2-iCre transgenic rats of both sexes. Intra-NAc infusions of Cre-inducible viral vectors containing stimulatory (hM3Dq) or inhibitory (hM4Di) designer receptors exclusively activated by designer drugs (DREADDs) were delivered after 4-weeks of alcohol intake, and clozapine-N-oxide (CNO) was administered to selectively manipulate NAc MSNs. Our results show that activation of NAc D1-MSNs increased alcohol intake 1-, 4-, and 24-h after the start of drinking while inhibition decreased it 1-h after the start of drinking, with no sex differences observed at any time point. Activation of NAc D2-MSNs had no impact on alcohol intake while inhibition increased alcohol intake in Drd2-iCre rats for 1-h in males and 4-h in females. These findings suggest opposing roles for how NAc D1- and D2-MSNs modulate alcohol intake in rats of both sexes.

## Introduction

Alcohol use disorder (AUD) is very prevalent, yet effective treatments are lacking since 75% of people with AUD relapse within 1 year^1^. Repeated cycles of compulsive alcohol use and withdrawal are hallmark characteristics of AUD that contribute to relapse and represent long-lasting aberrations in the neural circuitry responsible for reward and reinforcement^2,3^. In order to improve AUD treatment options, it is critical to gain a better understanding of the neuroadaptations that mediate these pathological behaviors.

The nucleus accumbens (NAc), a critical brain region involved in drug-seeking behavior, receives converging dopaminergic inputs from the ventral tegmental area, and glutamatergic input from the prefrontal cortex and amygdala^4,5^. The principal neurons within the NAc are GABAergic medium spiny neurons (MSNs), 90–95% of which contain either dopamine 1 receptors (D1-MSNs) or dopamine 2 receptors (D2-MSNs)^6–8^. Both MSN subtypes function through G-protein coupled processes in an opposing manner depending on their response to extracellular dopamine. D1 receptors couple to stimulatory G-proteins to facilitate production of cyclic AMP (cAMP), whereas D2 receptors couple to inhibitory G-proteins to inhibit cAMP production^9,10^. MSN subpopulations are further defined by their axonal projections. NAc D1-MSNs project either directly to the internal segment of the globus pallidus to disinhibit cortico-striatal and thalamic brain regions or indirectly through the ventral pallidum where they can either be involved in the activation or inhibition of these brain regions^11–13^. In general, NAc D1-MSNs are involved in facilitating motivated behaviors^14^. Conversely, NAc D2-MSNs indirectly project to the external segment of the globus pallidus through the ventral pallidum to inhibit these same outputs and facilitate negative reinforcement^11–13^.

Studies examining alcohol’s effects in the NAc have shown increased activation of D1-MSNs. For example, in male mice, alcohol intake increased protein expression of deltaFosB, a transcription factor considered a marker for addiction, exclusively in NAc D1-MSNs^15^. Acute alcohol exposure in male mice also enhanced glutamatergic transmission on NAc D1- but not D2-MSNs^16^. Furthermore, male mice chronically exposed to alcohol showed a shift from long-term depression to long-term potentiation exclusively in NAc D1-MSNs, suggesting alcohol sensitizes this MSN subpopulation^17–19^. Altogether, these studies suggest that enhanced excitation of NAc D1-MSNs may promote alcohol intake.

The role of NAc dopamine receptors in controlling alcohol intake in male mice have yielded conflicting results, which is likely because the techniques used targeted both cell-type specific postsynaptic dopamine receptors as well as presynaptic dopamine receptors that lack this cell-type specificity^20–24^. As such, the role of NAc D1- and D2-MSNs in controlling alcohol intake remains unexplored in either sex. Therefore, the main objective of this study was to investigate the role of NAc D1- versus D2-MSNs in mediating alcohol intake within transgenic male and female rats. Based on aforementioned evidence, we hypothesized that D1-MSNs in the NAc would promote alcohol intake. For this, Cre-inducible viral constructs containing either stimulatory (hM3Dq) or inhibitory (hM4Di) designer receptors exclusively activated by designer drugs (DREADDs) were infused into the NAc of Drd1a-iCre or Drd2-iCre male and female rats exposed to chronic, intermittent alcohol intake. The DREADDs agonist, clozapine-N-oxide (CNO) was used to selectively manipulate NAc MSNs, and the effect on alcohol intake was measured at 1, 4 and 24 h.

## Results

### Validation of DREADDs viral constructs in NAc D1- or D2 MSNs

**Figure 1:**
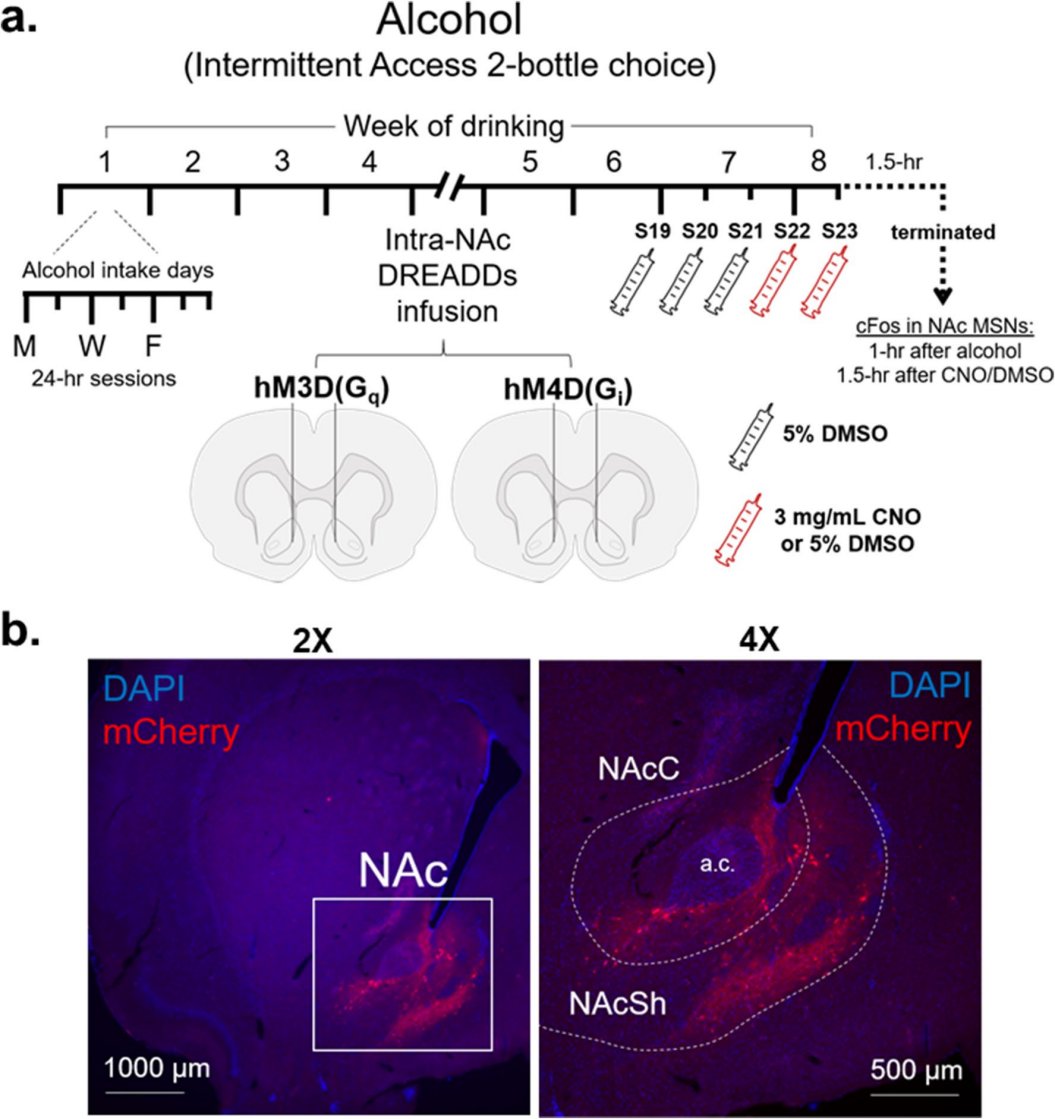
Experimental timeline and representative image of DREADDs expression in the NAc. (**a**) Drd1a-iCre, Drd2-iCre, and Wt rats underwent 4-weeks of the intermittent access to the IA2BC20% alcohol-drinking paradigm prior to receiving intra-NAc infusions of either the stimulatory hM3D(G_q_) or inhibitory hM4D(G_i_) DREADDs viral constructs. Rats continued on the IA2BC20% alcohol-drinking paradigm for 3-weeks; during week-7, all rats received intraperitoneal (i.p.) injections of 5% DMSO 30-min prior to drinking; on the first session of week-8 (session 22), rats received either i.p. injections of CNO (3 mg/mL) or 5% DMSO and were allowed access to alcohol for 24-h. On session 23, rats received another injection of either CNO or DMSO 30-min prior to the introduction of alcohol and water bottles and were terminated 1-h after the start of drinking and 90-min after i.p. injection of CNO or DMSO. Brains were collected to examine cFos in the NAc 90-min after CNO administration and 1-h after alcohol intake. (**b**) Representative image of DREADDs virus expression within the nucleus accumbens (NAc) at 2× (left) and 4× objective. Images display expression of the DREADDs fluorescent reporter, mCherry, against DAPI counterstain.

Prior to experimental testing, rats were genotyped to identify Drd1a-iCre, Drd2-iCre, and Wt rats (Supplemental Methods, Fig. S1). Adult rats began consuming alcohol intermittently for 8-weeks total, receiving bilateral intra-NAc infusions of either the hM3Dq or hM4Di DREADDs after 4-weeks of alcohol intake (Fig. 1a). On the final alcohol intake session, rats were terminated 90-min after i.p. administration of CNO or DMSO and 1-h after the start of alcohol intake; brains were immediately extracted and used for fluorescent in situ hybridization (FISH) or immunohistochemistry (IHC) techniques (Fig. 1a).

**Figure 2:**
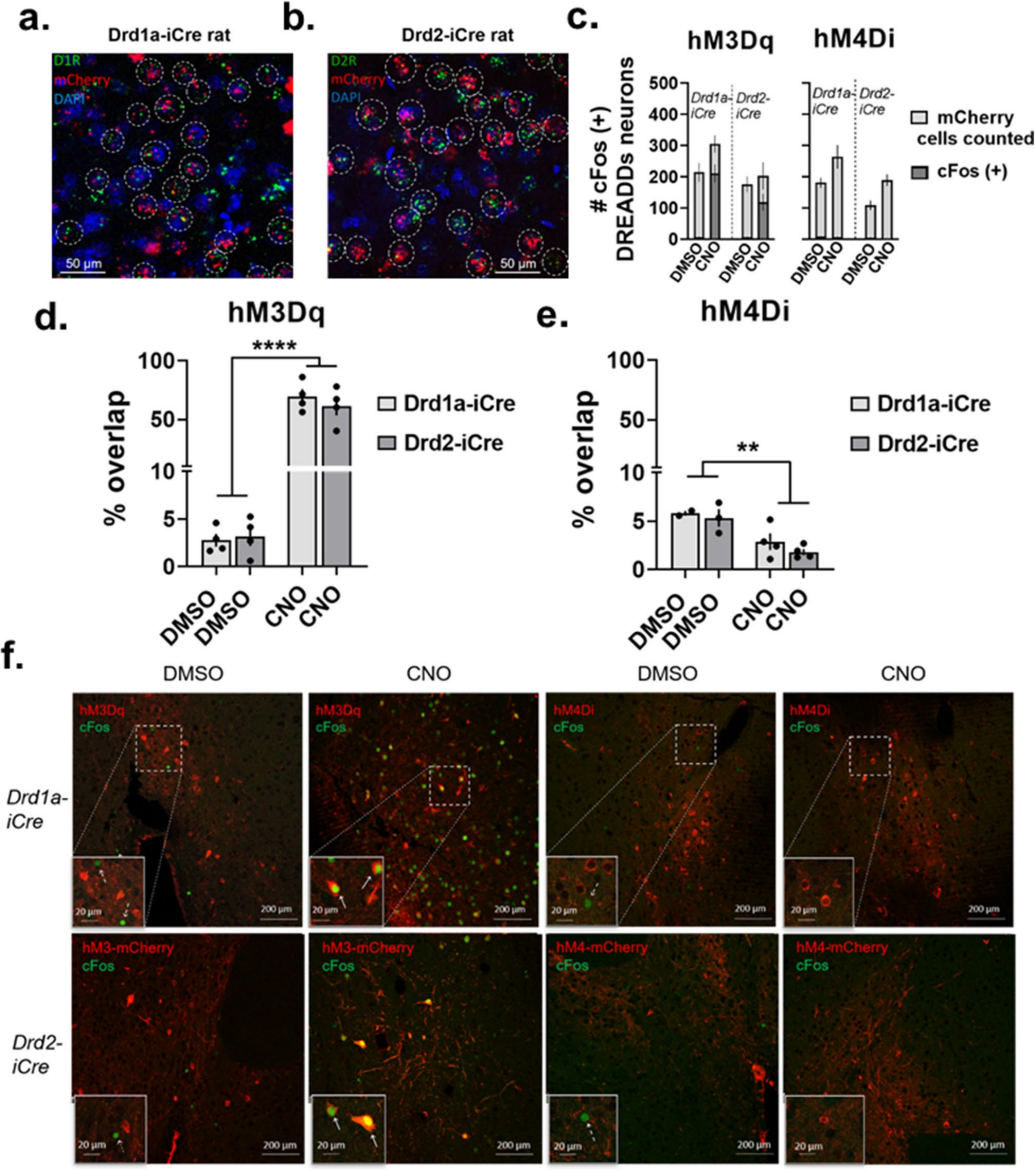
Validation of transgenic rats and DREADDs constructs. (**a**) Representative image showing co-labeling of Drd1a mRNA with mCherry, the fluorescent reporter fused to the Cre-dependent DREADDs construct, mRNA. (**b**) Representative image showing co-labeling of Drd2 mRNA with mCherry mRNA. (**a**,**b**) Images obtained using 63× objective, circles indicate co-labeled neurons. (**c**) Raw number of cFos-positive nuclei superimposed over the total number of mCherry-expressing neurons counted in rats with the hM3Dq DREADD (left) and hM4Di DREADD (right) (Drd1a-iCre_hM3Dq_DMSO = 214.3 ± 29.7, 6.3 ± 2.3; Drd1a-iCre_hM3Dq_CNO = 305 ± 27.3, 211.3 ± 23.8; Drd2-iCre_hM3Dq_DMSO = 176.5 ± 24.4, 6 ± 2.4; Drd2-iCre_hM3Dq_CNO = 203 ± 42.8, 119 ± 26.6; Drd1a-iCre_hM4Di_DMSO = 181 ± 16, 10.5 ± 0.5; Drd1a-iCre_hM4Di_CNO = 264 ± 37.5, 7 ± 1.8; Drd2-iCre_hM4Di_DMSO = 109 ± 15.1, 6 ± 1.5; Drd2-iCre_hM4Di_CNO = 189.5 ± 18.5, 3.5 ± 0.9). (**d**) In transgenic rats expressing the hM3Dq DREADD, CNO administration led to a significant increase in cFos-positive nuclei within mCherry-labeled neurons compared to DMSO administration. (**e**) In transgenic rats expressing the hM4Di DREADD, CNO reduced the percentage of cFos-positive nuclei in mCherry-labeled neurons compared to DMSO. (**f**) Representative images obtained with 20× objective of cFos and mCherry expression in the NAc of Drd1a-iCre (top) and Drd2-iCre (bottom) rats expressing either the hM3Dq (left two) or hM4Di (right two) DREADDs that were administered DMSO or CNO. Insets show representative cells zoomed in 10×; solid arrows indicate cFos-mCherry co-localization, dashed arrows indicate no co-localization. Data are represented as mean ± SEM, **p < 0.01, ****p < 0.0001. For IHC: 2–4 rats per group used, > 4 sections per rat were imaged, symbols represent average percent co-localization in the NAc of each rat brain imaged.

Transgenic rats were validated using FISH by examining co-labeling of Drd1a mRNA with mCherry, the fluorescent reporter fused to cre-dependent DREADDs, mRNA in Drd1a-iCre rats and co-labeling of Drd2 and mCherry mRNA in Drd2-iCre rats. In Drd1a-iCre and Drd2-iCre rats, mCherry is exclusively co-labeled on neurons tagged containing Drd1a or Drd2 mRNA, respectively (Fig. 2a,b; Figs. S2, S3). Together, this confirms that iCre is localized in D1-MSNs in Drd1a-iCre rats and D2-MSNs in Drd2-iCre rats. The hM3Dq and hM4Di DREADDs were validated using immunohistochemistry to examine cFos protein expression, a marker of neuronal activation, within mCherry-expressing neurons. Given that rats consumed alcohol for 1-h before being terminated, groups were balanced based on alcohol intake (g/kg) and two-way ANOVA comparing treatment and genotype revealed no significant interaction, suggesting rats administered DMSO vs. CNO consumed similar alcohol amount prior to examining cFos in the NAc (F_(1,25)_ = 1.48, p = 0.23, data not shown). Raw numbers of mCherry-labeled neurons and of cFos-positive nuclei that were counted are reported in Fig. 2c. The percentage of co-localization was calculated to normalize for differences in raw numbers between groups (Fig. 2d,e). In rats expressing the hM3Dq DREADD in the NAc, CNO increased cFos-positive nuclei within DREADDs-expressing neurons compared to rats expressing hM3Dq DREADD administered DMSO (two-way ANOVA, main effect of treatment: F_(1,12)_ = 147.6, p < 0.0001, Fig. 2d,f). In rats expressing the hM4Di DREADD in the NAc, CNO reduced the percentage of cFos-positive nuclei in mCherry-expressing neurons compared to the DMSO group (two-way ANOVA, main effect of treatment: F_(1,9)_ = 18.62, p = 0.0019, Fig. 2e,f).

### Alcohol intake and preference

**Figure 3:**
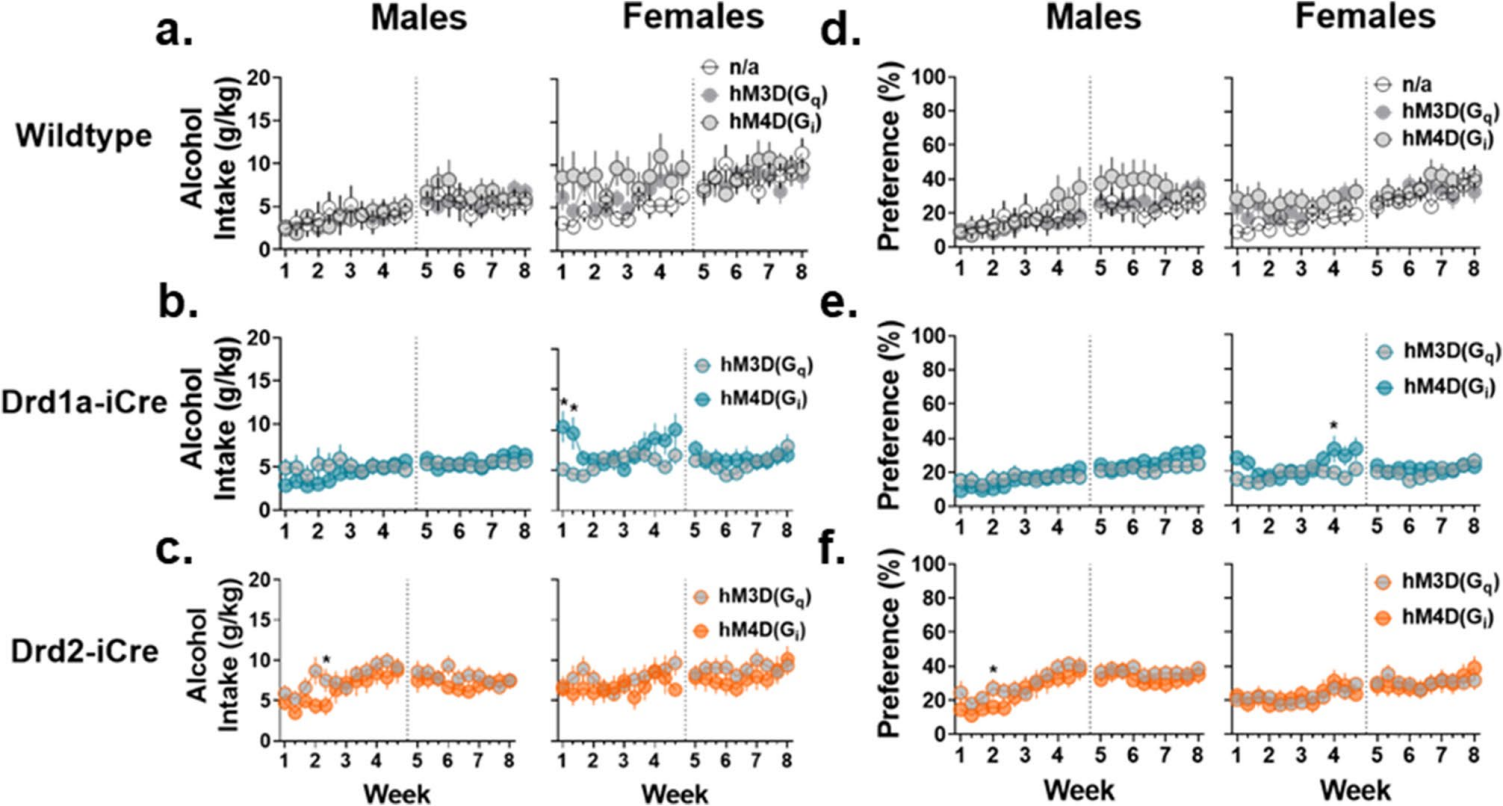
Alcohol intake and preference are unaffected by genotype or expression of DREADDs in the NAc in rats of either sex. (**a**) In Wt rats, groups that received either the hM3Dq, hM4Di, or no DREADD were compared to assess whether surgery impacted alcohol intake. Here, females displayed significantly increased alcohol intake (g/kg) compared to males but infusions of DREADDs constructs did not affect alcohol intake in either sex. (**b**) No differences were observed in Drd1a-iCre male rats. In female rats, the hM4Di group consumed significantly higher doses of alcohol during the first two sessions compared to the hM3Dq group. (**c**) Drd2-iCre male rats within the hM4Di group consumed more alcohol compared to the hM3Dq group on one session. No differences were observed in Drd2-iCre female rats. (**d**) In Wt rats receiving the hM3Dq, hM4Di, or no DREADDs infusion, percent preference for alcohol was similar throughout the 8-week drinking period. (**e**) Within Drd1a-iCre female rats, the hM4Di group displayed a significant increase in preference for alcohol compared to the hM3Dq group on session 10. No differences were observed in Drd1a-iCre males. (**f**) Within Drd2-iCre male rats, the hM4Di group displayed a significant increase in preference for alcohol compared to the hM3Dq group during session 4. Data are represented as mean ± SEM, *p < 0.05.

Alcohol intake (g/kg) and preference (%) were measured throughout the 8-week period and analyzed to assess whether they were affected by sex, genotype, DREADDs constructs and time. Additionally, body weights, weight gain, total fluid intake, and food intake were also examined between sexes and are reported in Fig. S4. For Wt groups, rats received infusions of either the hM3Dq or hM4Di DREADDs into the NAc, while one group received no surgery and was used to examine whether surgery impacted alcohol intake. In Wt rats, three-way linear mixed model (LMM) ANOVA revealed a main effect of sessions and sex, indicating that rats escalate alcohol intake over the 8-week period and that Wt female rats consume more alcohol than Wt males (effect of sessions: F_(21,858)_ = 6.71, p < 0.0001; sex: F_(1,42)_ = 10.34, p = 0.0025, Fig. 3a). Alcohol intake was then analyzed between Wt, Drd1a-iCre, and Drd2-iCre rats that received infusions of either the hM3Dq or hM4Di DREADDs to investigate whether these constructs impacted alcohol intake. Four-way LMM ANOVA revealed a significant interaction between sex, genotype, DREADDs, and session when examining alcohol intake over the 8-week period (interaction: F_(42,2856)_ = 1.59, p = 0.009). In Drd1a-iCre female but not male rats, significant differences in alcohol intake were observed between the hM3Dq and hM4Di groups, though these differences occurred prior to receiving DREADDs infusions (Tukey’s post hoc: sessions 1–2: t_(141)_ = − 3.09, − 2.97, p = 0.0024, 0.0035, Fig. 3b). Similarly, in Drd2-iCre male but not female rats, significant differences in alcohol intake were observed between the hM3Dq and hM4Di groups, but these differences also occurred prior to receiving DREADDs infusions (Tukey’s post hoc: t_(141)_ = 2.28, p = 0.024, Fig. 3c). Overall, these results suggest that female rats display higher alcohol intake than males but that neither genotype nor DREADDs infusions impact this.

Within Wt rats receiving either the hM3Dq, hM4Di, or no DREADDs, three-way LMM ANOVA revealed an interaction between sex, DREADDs, and session but Tukey’s post hoc showed no significant between-group differences when analyzing percent preference (F_(42,882)_ = 1.53, p = 0.017, Fig. 3d). Percent preference was then analyzed comparing Wt, Drd1a-iCre, and Drd2-iCre rats expressing either the hM3Dq or hM4Di DREADD a significant four-way interaction was observed (F_(42,2857)_ = 1.61, p = 0.0082, Fig. 3e). Tukey’s post hoc revealed that within Drd1a-iCre female rats, the hM4Di group displayed increased percent preference for alcohol on session 10 compared to the hM3Dq group (t_(141)_ = − 2.04, p = 0.04, Fig. 3e). Furthermore, in Drd2-iCre male rats, the hM4Di group displayed increased percent preference compared to the hM3Dq group on session 4 (t_(141)_ = 2.001, p = 0.047, Fig. 3f). Importantly, significant differences observed in alcohol preference occurred prior to DREADDs infusions, suggesting that the infusions themselves do not impact alcohol preference. Furthermore, no main effects of sex were observed for alcohol preference, suggesting male and female rats similarly prefer alcohol when body weight is normalized.

### Effects of CNO on wildtype control rats

**Figure 4:**
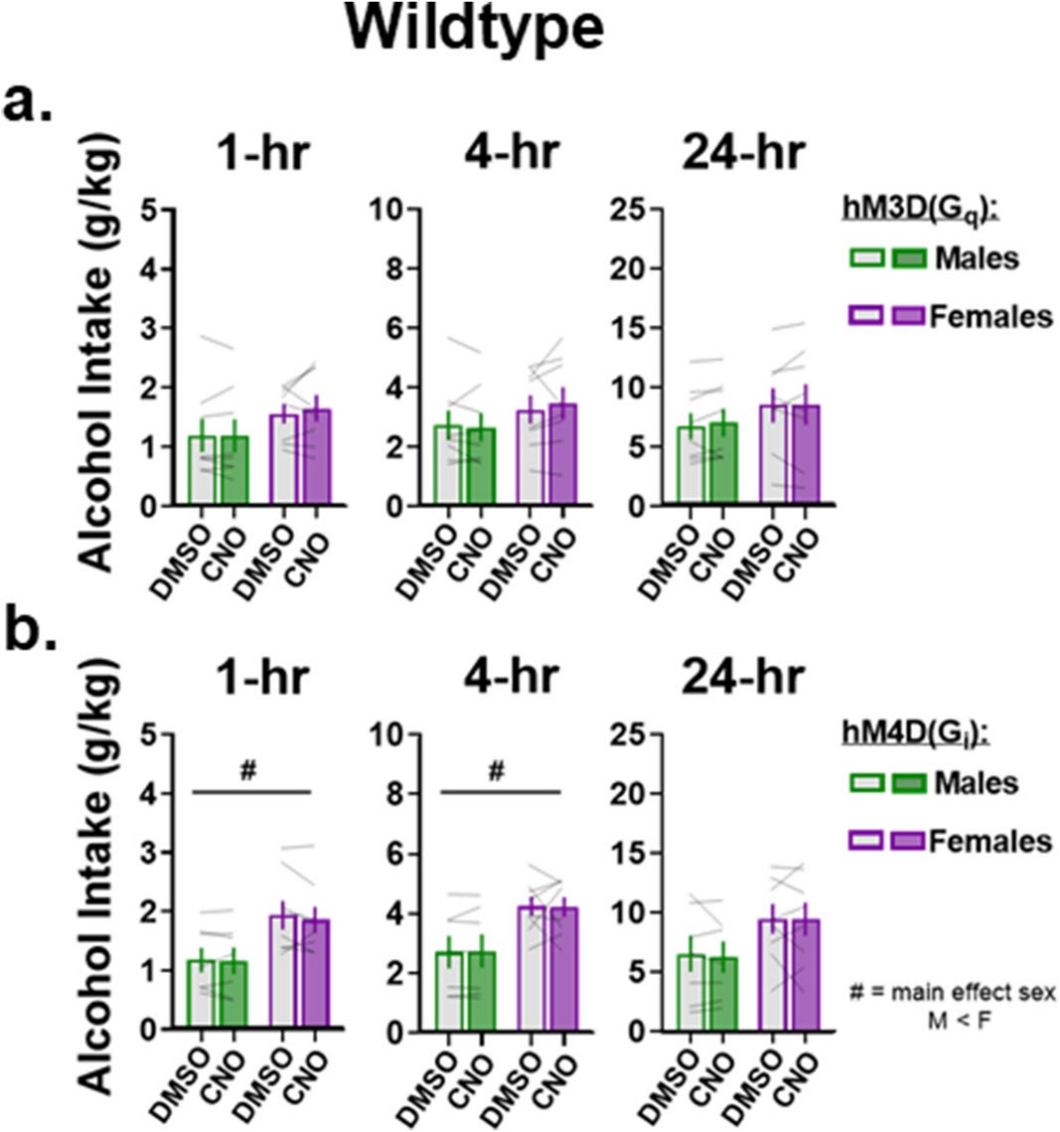
CNO does not affect alcohol intake in wildtype rats of either sex. (**a**) In Wt rats that received intra-NAc infusions of the hM3Dq DREADD, CNO did not impact alcohol intake at any time point measured in either sex. (**b**) In Wt rats that received intra-NAc infusions of the hM4Di DREADD, CNO did not impact alcohol intake. Females consumed more alcohol overall compared to males 1- and 4-h after the start of alcohol intake. Data are represented as mean ± SEM, # = main effect of sex, p < 0.05.

Based on a previous report suggesting that systemic administration of CNO can enter the brain as back-converted clozapine, controlling for any effects this might have on alcohol intake was warranted^25^. To do this, CNO was administered to Wt rats and alcohol intake after CNO administration was compared to the same rats’ alcohol intake from the week prior when receiving DMSO injections. Given that within-subjects effects were examined, repeated measures (RM) two-way mixed effects ANOVA was used to assess whether alcohol intake is altered after CNO within each rat and whether any sex differences exist. In Wt rats that received hM3Dq, no effects of treatment nor sex were observed with RM two-way ANOVA, suggesting that CNO does not impact alcohol intake in these rats (sex x treatment interaction: 1-h: F_(1,14)_ = 0.62, p = 0.45; 4-h: F_(1,14)_ = 0.75, p = 0.4; 24-h: F_(1,14)_ = 0.21, p = 0.65, Fig. 4a). In Wt rats that received hM4Di, RM two-way ANOVA revealed a main effect of sex 1- and 4-h, but not 24-h, after the start of drinking with no effect of treatment, suggesting that females consumed more alcohol than males in the beginning of the drinking session but that CNO does not alter alcohol intake in either sex (main effect of sex, 1-h: F_(1,13)_ = 5.3, p = 0.039; 4-h: F_(1,13)_ = 6.44, p = 0.025; 24-h: F_(1,13)_ = 2.62, p = 0.13, Fig. 4b). Together, these data indicate that CNO by itself does not impact alcohol intake in rats of either sex.

### Effects of chemogenetic manipulation of NAc D1R-containing MSNs on alcohol intake

**Figure 5:**
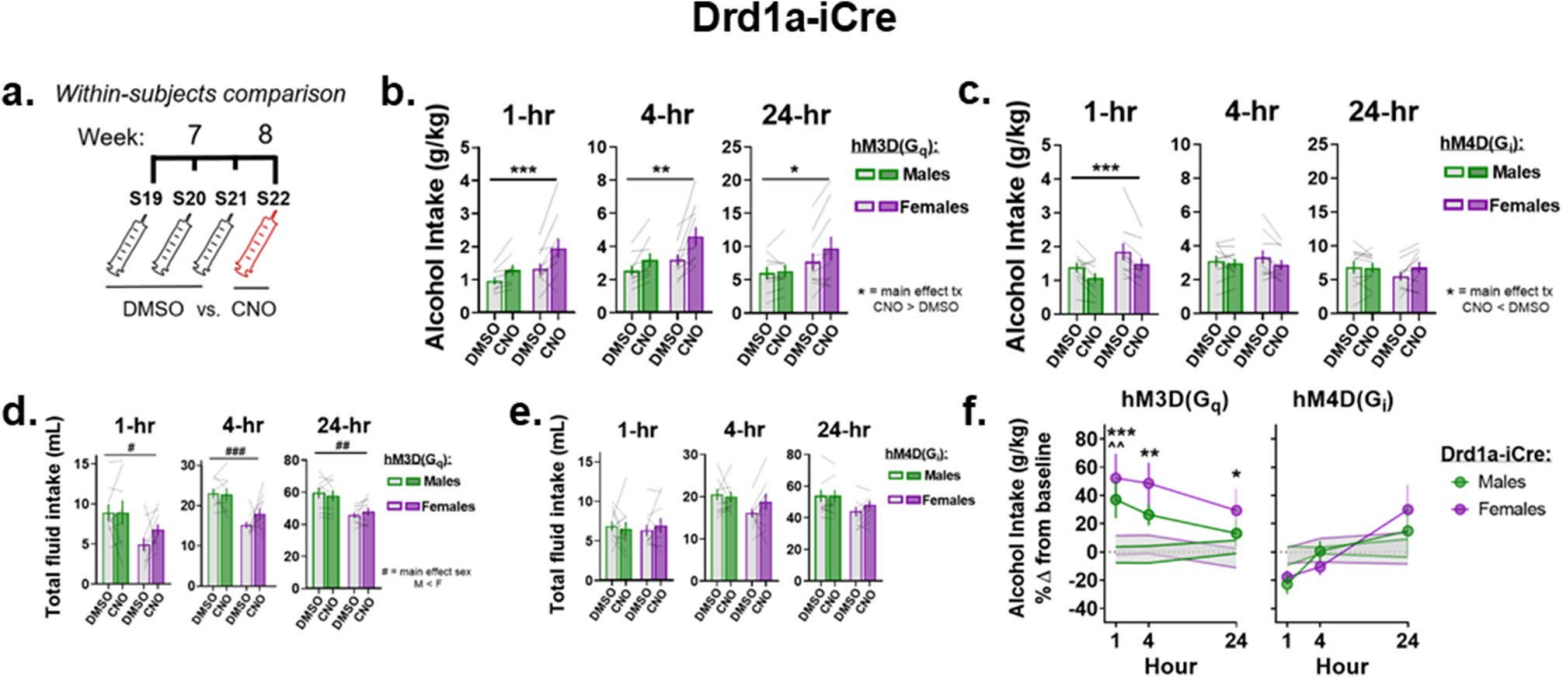
NAc D1-MSNs bidirectionally control alcohol intake within Drd1a-iCre rats of both sexes. (**a**) Timeline of DMSO and CNO administration. Sessions 19–21: DMSO was administered 30-min before the start of each alcohol session, and bottles were weighed 1-, 4-, and 24-h after the start of drinking. Session 22: CNO was administered to the same rats that received DMSO during sessions 19–21 30-min before the start of drinking, and bottles were weighed 1-, 4-, and 24-h later. (**b**) CNO administration to Drd1a-iCre rats expressing the hM3Dq DREADD increased alcohol intake 1-, 4-h, and 24-h after the start of alcohol-drinking compared to when the same rats were administered DMSO during week-7. (**c**) Drd1a-iCre rats expressing the hM4Di DREADD displayed a reduction in alcohol intake 1-h after the start of drinking when administered CNO compared to DMSO. (**d**,**e**) Total fluid intake after CNO administration compared to total fluid intake after DMSO administration was unaltered regardless of time and DREADDs virus. Drd1a-iCre male rats expressing hM3Dq showed significantly increased total fluid intake compared to females. (**f**, left.) When collapsed by sex, Drd1a-iCre rats with the hM3Dq DREADD displayed significantly increased percent changes in alcohol intake compared to Wt rats that received the hM3Dq DREADD infusion across all three time points measured. When separated by sex, this increase was apparent in Drd1a-iCre males 1-h after the start of drinking compared to 1-, 4-, and 24-h in Drd1a-iCre females. (**f**, right.) No significant percent changes in alcohol intake were observed when comparing Drd1a-iCre rats expressing hM4Di to Wt rats that received hM4Di. Data represented as mean ± SEM (**b**–**e**) main effect of treatment *p < 0.05, **p < 0.01, ***p < 0.001; main effect of sex ^#^p < 0.05, ^##^p < 0.01, ^###^p < 0.001. (**f**) Females *p < 0.05, **p < 0.01, ***p < 0.001; Males ^^^^p < 0.01.

Alcohol intake after CNO administration was compared to alcohol intake after DMSO administration within the same Drd1a-iCre rats (see timeline, Fig. 5a). In Drd1a-iCre rats expressing the hM3Dq DREADD, RM two-way ANOVA revealed no significant interaction or main effect of sex, though a significant main effect of treatment was observed at each time point measured (1-, 4-, and 24-h), suggesting that CNO significantly increased alcohol intake compared to the same rats’ alcohol intake after DMSO administration during week-7 (main effect of treatment: 1-h: F_(1,17)_ = 19.05, p = 0.0004; 4-h: F_(1,17)_ = 15.72, p = 0.001; 24-h: F_(1,17)_ = 6.79, p = 0.019, Fig. 5b). In Drd1a-iCre rats expressing hM4Di, RM two-way ANOVA showed no significant interactions or main effects of sex, but a main effect of treatment was observed 1-h, but not 4- and 24-h, after the start of drinking, suggesting that alcohol intake after CNO administration is reduced compared to intake after DMSO in Drd1a-iCre rats expressing hM4Di (1-h: F_(1,18)_ = 20.06, p = 0.0003; 4-h: F_(1,18)_ = 3.89, p = 0.06; 24-h: F_(1,18)_ = 2.8, p = 0.11, Fig. 5c).

Total fluid intake was unaffected by CNO administration across all time points in Drd1a-iCre rats expressing hM3Dq, though two-way RM ANOVA showed a significant main effects of sex in Drd1a-iCre rats expressing hM3Dq 1-, 4-, and 24-h after the start of drinking, suggesting that male rats consumed more total fluid compared to females (main effect of sex, 1-h: F_(1,17)_ = 5.78, p = 0.03; 4-h: F_(1,17)_ = 22.91, p = 0.0002; 24-h: F_(1,17)_ = 10.85, p = 0.004, Fig. 5d). Furthermore, RM two-way ANOVA revealed no differences in total fluid intake among Drd1a-iCre rats expressing hM4Di (sex × treatment: 1-h: F_(1,18)_ = 0.69, p = 0.42; 4-h: F_(1,18)_ = 2.31, p = 0.15; 24-h: F_(1,18)_ = 2.19, p = 0.16, Fig. 5e). Taken together, these data indicate that selectively activating D1R-containing NAc MSNs increases alcohol intake while inhibiting D1R-containing MSNs decreases alcohol intake.

The percent change in alcohol intake after CNO administration compared to DMSO administration was examined across each time point to both control for between-subject variability in the raw alcohol intake data and to compare between-genotype effects. Four-way LMM ANOVA revealed a significant genotype × DREADDs × session interaction (F_(2,118)_ = 5.36, p = 0.0059, Fig. 5f). Tukey’s post-hoc analysis showed that when collapsed by sex, Drd1a-iCre rats expressing the hM3Dq DREADD showed significantly increased percent changes in alcohol intake compared to Wt rats that received intra-NAc infusions of hM3Dq and CNO administration 1-, 4-, and 24-h after the start of drinking (hM3Dq, Drd1a-iCre vs. Wt: 1-h: t_(63)_ = 4.25, p = 0.0001; 4-h: t_(63)_ = 3.42, p = 0.001; 24-h: t_(63)_ = 2.19, p = 0.032, Fig. 5f, left). Additionally, no between-genotype differences were observed in Drd1a-iCre rats expressing hM4Di compared to Wt rats when examining percent change in alcohol intake collapsed by sex (Tukey’s post hoc, hM4Di, Drd1a-iCre vs Wt: 1-h: t_(63)_ = − 1.78, p = 0.08; 4-h: t_(63)_ = − 0.51, p = 0.61; 24-h: t_(63)_ = 1.395, p = 0.17, Fig. 5f, right). To better understand whether the percent increases in alcohol intake observed in Drd1a-iCre rats expressing the hM3Dq DREADD were driven by males or females, a priori hypothesis testing was used to run separate post hoc analyses within each sex. In rats that received the hM3Dq DREADD, Drd1a-iCre males showed significantly increased percent changes in alcohol intake 1-h after the start of drinking compared to Wt males, while female Drd1a-iCre rats expressing hM3Dq showed significant increases compared to Wt throughout the 24-h period (Tukey’s post hoc within-sex for Drd1a-iCre vs Wt: Males, 1-h: t_(29)_ = 3.19, p = 0.0034; 4-h: t_(29)_ = 1.99, p = 0.056; 24-h: t_(29)_ = 0.702, p = 0.49; Females: 1-h: t_(30)_ = 2.76, p = 0.0097; 4-h: t_(30)_ = 2.59, p = 0.015; 24-h: t_(30)_ = 2.11, p = 0.044, Fig. 5f left). Taken together, these data indicate that activation of NAc D1-MSNs promotes alcohol intake in Drd1a-iCre rats of both sexes.

### Effects of chemogenic manipulation of NAc D2R-containing MSNs on alcohol intake

**Figure 6:**
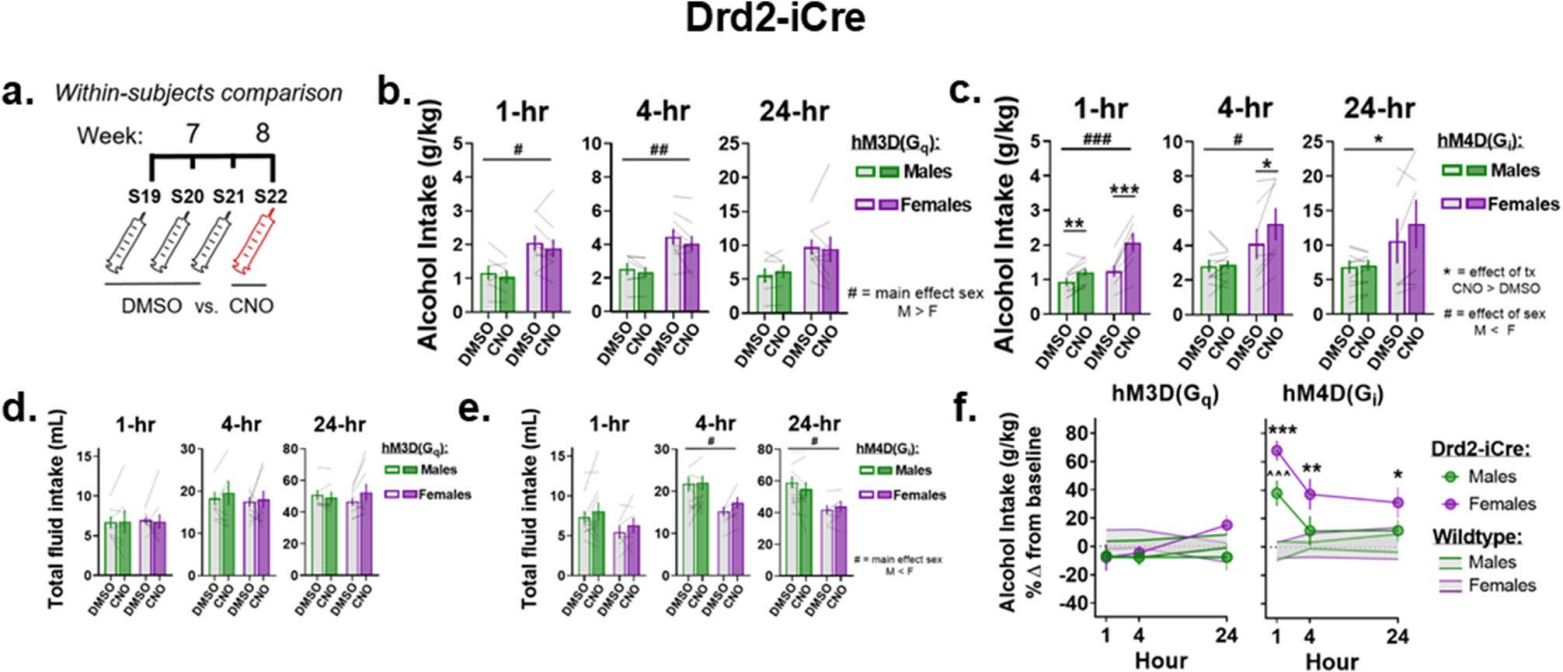
Inhibition of NAc D2-MSNs in Drd2-iCre rats promotes alcohol intake. (**a**) Timeline of DMSO and CNO administration. Sessions 19–21: DMSO was administered 30-min before the start of each alcohol session, and bottles were weighed 1-, 4-, and 24-h after the start of drinking. Session 22: CNO was administered to the same rats that received DMSO during sessions 19–21 30-min before the start of drinking, and bottles were weighed 1-, 4-, and 24-h later. (**b**) Drd2-iCre rats expressing hM3Dq DREADD showed no change in alcohol intake after CNO administration across all time points. Females drank significantly more alcohol than males at 1- and 4-h after the start of drinking. (**c**) Drd2-iCre male rats expressing the hM4Di DREADD displayed a significant increase in alcohol intake 1-h after CNO administration. Drd2-iCre female rats with the hM4Di DREADD displayed a significant increase in alcohol intake 1- and 4-h after CNO administration. CNO increased alcohol intake overall at the 24-h time point. Drd2-iCre females expressing hM4Di drank significantly more alcohol after CNO administration, but not DMSO, 1- and 4-h after the start of alcohol drinking. (**d**,**e**) CNO administration did not alter total fluid intake across any time point in Drd2-iCre rats compared to total fluid intake after DMSO administration. (**f**, left.) Drd2-iCre rats with the hM3Dq DREADD were no different than Wt rats for percent change in alcohol intake after CNO. (**f**, right.) Collapsed by sex, Drd2-iCre rats expressing hM4Di showed significant increases in percent change in alcohol intake compared to Wt receiving hM4Di 1- and 4-h after the start of alcohol-drinking. In male rats, Drd2-iCre showed significant increases in percent change in alcohol intake compared to Wt with the hM4Di DREADD 1-h after the start of drinking. Increased percent change in alcohol intake after CNO among Drd2-iCre female rats expressing hM4Di persisted for 24-h after the start of alcohol intake compared to Wt with hM4Di. Data represented as mean ± SEM. (**b**–**e**) treatment effects *p < 0.05, **p < 0.01, ***p < 0.001; sex effects ^#^p < 0.05, ^##^p < 0.01, ^###^p < 0.001. (**f**) Females *p < 0.05, **p < 0.01, ***p < 0.001; Males ^^^p < 0.001.

Alcohol intake after CNO administration was compared to alcohol intake after DMSO administration within the same Drd2-iCre rats (see timeline, Fig. 6a). In Drd2-iCre rats expressing the hM3Dq DREADD, two-way RM ANOVA revealed that CNO had no effect on alcohol intake compared to when rats were administered DMSO, though a significant main effect of sex was observed at the 1- and 4-h time points, suggesting that female rats consumed more alcohol than males (main effect of sex, 1-h: F_(1,14)_ = 7.41, p = 0.02; 4-h: F_(1,14)_ = 9.3, p = 0.009; 24-h: F_(1,14)_ = 3.8, 0.072, Fig. 6b). In Drd2-iCre rats expressing hM4Di, significant treatment x sex interactions were revealed when analyzing the 1- and 4-h measurements (interaction, 1-h: F_(1,16)_ = 20.2, p = 0.0004; 4-h: F_(1,16)_ = 6.97, p = 0.018; 24-h: F_(1,16)_ = 4.4, p = 0.052, Fig. 6c). Sidak’s post-hoc analysis revealed that CNO led to a significant increase in alcohol intake compared to DMSO in Drd2-iCre rats expressing hM4Di in both males and females 1-h after the start of drinking, but only in female rats 4-h after the start of drinking (Males: 1-h: t_(16)_ = 3.54, p = 0.005, 4-h: t_(16)_ = 0.34, p = 0.93 ; Females, 1-h: t_(16)_ = 8.58, p < 0.0001, 4-h: t_(16)_ = 2.96, p = 0.018, Fig. 6c). Additionally, analysis on the 1- and 4-h time point data revealed that Drd2-iCre female rats expressing hM4Di displayed higher levels of alcohol intake compared to males when administered CNO but not DMSO (M v F, CNO, 1-h: t_(32)_ = 3.87, p = 0.001; 4-h: t_(32)_ = 2.9, p = 0.013, Fig. 6c). Though no interaction was observed during the 24-h time point, a significant main effect of treatment was observed following two-way RM ANOVA for alcohol intake after CNO compared to DMSO (F_(1,16)_ = 6.37, p = 0.02, Fig. 6c).

CNO did not impact total fluid intake in Drd2-iCre rats expressing either hM3Dq or hM4Di, though a main effect of sex was observed in the hM4Di group at the 4- and 24-h time points, suggesting males consumed more total fluid than females at these times (two-way RM ANOVA, effect of sex, hM4Di, 4-h: F_(1,16)_ = 6.13, p = 0.025; 24-h: F_(1,16)_ = 6.1, p = 0.025, Fig. 6d,e). Taken together, these data indicate that activation of NAc D2-MSNs has no effect on alcohol intake while inhibition of NAc D2-MSNs promotes alcohol intake. Furthermore, Drd2-iCre female rats expressing hM4Di consumed significantly more alcohol than males after CNO, but not DMSO, suggesting that females may be more sensitive to the effect that inhibition of NAc D2-MSNs has on alcohol intake.

To control for within-group variability in raw alcohol intake (g/kg) and examine between-genotype differences, the percent change in alcohol intake after CNO was calculated and values were compared between Drd2-iCre and Wt rats that received comparable DREADDs viruses. Four-way LMM ANOVA revealed a significant genotype × DREADDs × session interaction (F_(2,116)_ = 4.91, p = 0.009, Fig. 6f). For Drd2-iCre rats expressing the hM3Dq DREADD, percent change in alcohol intake after CNO was no different from Wt receiving hM3Dq when data was collapsed by sex (Tukey’s post hoc, 1-h: t_(62)_ =  − 0.74, p = 0.46; 4-h: t_(62)_ =  − 0.65, p = 0.52; 24-h: t_(62)_ = 0.34, p = 0.74, Fig. 6f, left). For Drd2-iCre rats expressing hM4Di, Drd2-iCre rats showed significantly higher percent changes in alcohol intake compared to Wt rats with the hM4Di DREADD 1- and 4-h after the start of alcohol-drinking (Tukey’s post hoc, 1-h: t_(62)_ = 5.07, p < 0.0001; 4-h: t_(62)_ = 3.16, p = 0.0024; 24-h: t_(62)_ = 1.904, p = 0.062, Fig. 6f, right). A priori hypothesis testing was used to examine percent increase changes in alcohol intake separately within each sex of Drd2-iCre rats expressing the hM4Di DREADD. Tukey’s post hoc showed that Drd2-iCre male rats displayed significantly increased percent changes in alcohol intake compared to Wt males that received hM4Di 1-h after the start of drinking, though this effect dissipated at 4- and 24-h into the session (Tukey’s post hoc, 1-h: t_(29)_ = 3.8, p = 0.0007; 4-h: t_(29)_ = 0.97, p = 0.34; 24-h: t_(29)_ = 0.83, p = 0.42, Fig. 6f, right). Within Drd2-iCre females, however, the percent increases in alcohol intake from CNO remained significantly higher compared to Wt throughout the 24-h session (Tukey’s post hoc, 1-h: t_(29)_ = 4.24, p = 0.0002; 4-h: t_(29)_ = 3.72, p = 0.0008; 24-h: t_(29)_ = 2.05, p = 0.049, Fig. 6f, right). Altogether, these data indicate that inhibition of NAc D2R-MSNs promotes alcohol intake within Drd2-iCre rats of both sexes.

## Discussion

This study aimed to examine the roles of NAc D1- versus D2-MSNs in modulating alcohol intake in rats of both sexes. Activation of NAc D1-MSNs increased alcohol intake when comparing both within-subject and between-genotypes. Inhibition of NAc D1-MSNs reduced alcohol intake when making within-subjects comparisons but not when comparing to Wt controls, and activation of D2-MSNs had no impact on alcohol intake. Inhibition of NAc D2-MSNs increased alcohol intake in rats of both sexes, an effect that was greater in females when comparing within-subjects. Selective activation or inhibition of NAc D1- or D2-MSNs was verified by demonstrating increased or decreased cFos colocalization within hM3Dq or hM4Di neurons, respectively, in rats administered CNO as compared to those given DMSO.

In general, functional studies using chemogenetic approaches have demonstrated that inhibition of NAc MSNs reduces while activation of NAc MSNs increases alcohol intake in rats and mice^26–28^. Pharmacological studies suggest that these effects may be facilitated through D1Rs given that D1R antagonism inhibits while D1R agonism in the NAc enhances alcohol-seeking behaviors^29,30^. Additionally, studies examining the role of D2Rs have yielded conflicting reports since some found that overexpression of D2Rs in the NAc reduced alcohol intake while another found no effects^20,22–24^. It should be noted, however, that the studies mentioned above did not selectively target D1- versus D2-MSNs but rather all dopamine D1 or D2 receptors in the NAc, which affect both pre- and postsynaptic terminals^31,32^.

Electrophysiological studies examining functional changes on NAc D1-MSNs following alcohol exposure suggest increased potentiation of this MSN subtype. Specifically, acute alcohol exposure increased glutamatergic transmission on NAc D1- but not D2-MSNs in Drd1-Cre male mice^16^. Furthermore, chronic alcohol exposure led to a shift from LTD to LTP exclusively in D1R-MSNs of male mice, suggesting a heightened potentiation within this MSN subtype^17–19^. These findings parallel those from similar studies examining glutamatergic transmission on D1-MSNs in the dorsomedial striatum (DMS) following chronic alcohol exposure, which is enhanced through increased AMPA receptor activity^33,34^. Furthermore, activation of D1-MSNs in DMS increased alcohol intake while inhibition reduced it in male mice^34^, suggesting a role of DMS D1-MSNs in bidirectionally controlling alcohol intake. The data from the present study extended these findings to the NAc for the first time in either sex by showing that D1-MSNs promote alcohol intake since activation increased alcohol intake while inhibition reduced it in Drd1a-iCre rats. It should be noted, however, that inhibition of NAc D1-MSNs only modestly reduced alcohol intake given that the effects observed were significant when comparing within-subjects changes but not when comparing percent changes in alcohol intake in Drd1a-iCre versus Wt rats, a comparison that was made to account for natural fluctuations in alcohol intake among Wt control rats. Given that the role of D1-MSNs in alcohol intake remained unexplored in females, this study extended these findings to this sex for the first time by showing that D1R-containing NAc MSNs positively regulate alcohol intake in Drd1a-iCre rats of both sexes.

Conversely, multiple reports have shown that NAc D2R-containing MSNs do not change their physiology following alcohol exposure. In the Beckley et al*.* study mentioned above, acute alcohol did not affect glutamatergic transmission on D2R-containing MSNs within the NAc of Drd1-Cre male mice. Similarly, chronic alcohol exposure did not impact excitability of D2R-containing MSNs within the NAc of male mice^17,18^. These studies are in line with a previous report showing that NAc protein expression of deltaFosB was unchanged on D2-MSNs following chronic alcohol intake^15^. Together, these findings suggest that D2-MSNs in the NAc may not play a direct role in modulating alcohol intake. This contrasts with studies examining the DMS, which have shown that alcohol intake is facilitated through the inhibition of D2-MSNs and inhibited through the activation of D2-MSNs^34^. In the current study, there was no observable effect on alcohol intake after activating NAc D2-MSNs, but significant increases in alcohol intake were revealed following inhibition of D2-MSNs in both sexes. Interestingly, the increase in alcohol intake as a result of inhibiting NAc D2-MSNs occurred to a higher degree and for a prolonged period in Drd2-iCre female as compared to male rats. Based on these findings as well as the studies mentioned above, we propose that the NAc and DMS differ in their control of alcohol intake and that while D1-MSNs in the NAc directly control alcohol intake, D2-MSNs may have an indirect role given that only inhibition of D2-MSNs altered alcohol intake. It is, therefore, possible that the increase in alcohol intake following inhibition of D2R-MSNs in the NAc is likely due to increased potentiation of D1R-MSNs as a compensatory, indirect mechanism.

It should be noted, however, that the present study targeted NAc D2Rs on all neuronal subtypes and while the majority (95%) are MSNs, cholinergic interneurons also express D2Rs^35^. Although they represent a small population of NAc neurons (2%), NAc cholinergic interneurons may have played a role in the D2R-mediated changes in alcohol intake^35–37^.

While the role of NAc D1-MSNs in alcohol intake was similar between male and female rats, the increase in alcohol intake following inhibition of NAc D2-MSNs was greater and more prolonged in female rats. Inhibition of D2-MSNs in female rats increased alcohol intake to a greater degree in females compared to males 1- and 4-h after the start of drinking. Additionally, while this effect diminished in males after 1-h, the effects persisted in females at the 4-h measurement. It is possible that metabolism of CNO is slower in females. If so, prolonged changes in alcohol intake may be explained as an artifact of the chemogenetic manipulation rather than sex differences in how NAc MSN subtypes regulate alcohol intake. While a previous report detailed metabolism of CNO across a wide range of doses in male rats and mice, this information remains unknown for female rodents^25^. A report in humans showed no differences in CNO metabolism between men and women^38^. Furthermore, if CNO metabolism differences contributed to these effects, the changes would be observed in Drd1a-iCre rats as well. Additionally, studies using equal doses of CNO to activate DREADDs in male and female rodents did not find increased sensitivity to CNO or prolonged effects on behavior among females^39–41^. It is also worth noting that, in the present study, CNO administration did not impact alcohol intake in male or female Wt rats, providing further evidence that the CNO-mediated effects on alcohol intake are specific to NAc MSN cell-type manipulations.

It is possible that NAc D2-MSNs modulate alcohol’s addictive properties differently between the sexes. In fact, a recent report showed dynamic changes in NAc MSN excitatory synaptic input and intrinsic excitability during different estrous cycle phases, indicating the presence of ovarian hormones may heighten NAc excitability^42^. Interestingly, one report showed that D2R availability increased in the presence of progesterone and estrogen, suggesting a potentially enhanced sensitivity to the inhibitory effect on NAc D2-MSNs in female rats^43^. As such, it is possible that the presence of ovarian hormones prolonged the observed changes in alcohol intake in female rats.

In conclusion, or data demonstrate a clear relationship between NAc D1- and D2-MSNs in modulating alcohol intake rats of both sexes. Activation of D1-MSNs increased while inhibition decreased alcohol intake in rats of both sexes. Inhibition of D2-MSNs in the NAc also increased alcohol intake, likely through an indirect mechanisms. Overall, these findings will shed more light on the neuroadaptations that regulate alcohol’s addictive properties for both men and women.

## Methods

### Animals and housing

Male and female transgenic Long Evans Drd1a-iCre (LE-Tg(Drd1a-iCre)3Ottc) and Drd2-iCre (LE-Tg(Drd2-iCre)1Ottc) rats were obtained from the National Institute on Drug Abuse transgenic animal core facility (NIDA transgenic rat project; Rat Resource and Research Center (RRRC), Columbia, MO) and crossed with wild type (Wt) Long-Evans (Charles River, Wilmington, MA) as previously described^44,45^. Drd1a-iCre rats contain a 1.3 kb cassette containing *iCre* located on the promoter region of the rat gene for the dopamine 1 receptor, *Drd1a* (CH230-115J9) and are entered into the Rat Genome Database (RGD: 10412325) and deposited at the RRRC as #767^45^. Drd2-iCre rats contain a 1.3 kb cassette containing *iCre* located on the promoter region of the rat gene for the dopamine 2 receptor (CH230-11B15) and are in the Rat Genome Database (RGD: 10412327) and deposited at the RRRC as #768 (NIDA Transgenic Rat Project, RRRC).

A breeding colony was maintained to produce Drd1a-iCre and Drd2-iCre heterozygous male and female rats in a temperature- and humidity-controlled room under a 12-h light/dark cycle (8a-8p). Rat pups were weaned 21-days after birth and were pair-housed with same-sex littermates until being transferred to a separate room 1 week prior to the start of experimental procedures. This 1-week period allowed rats to adapt to a reverse light/dark cycle (lights off: 11 a.m.; lights on: 11 p.m.) which was maintained throughout the course of the 8-week study. Given that alcohol drinking procedures occurred in the home cage, 7-week old rats were single-housed in 43 × 21.5 × 25.5 cm Plexiglass cages with environmental enrichment consisting of one 4-in. PVC pipe to reduce the effects of social-isolation stress. Rats were 8 weeks old at the start of experimental procedures, corresponding to 225–250 g body mass for female rats and 325–375 g for male rats. A total of 197 rats were used in the current study (100 male and 97 female rats); 2 male rats were removed from the study after having to be terminated for unknown illnesses that caused them to lose > 15% of their body weight; 12 rats (6 males, 6 females) were removed from the study for having expression of designer receptors activated exclusively by designer drugs (DREADDs) viral constructs outside of the NAc. In the current study, 99 transgene-positive rats were terminated via intracardial perfusion and 12 were removed for having DREADDs virus expressed outside of the NAc. Of the 87 remaining, 80/87 (91.9%) showed DREADDs viral construct expression in both the core and shell subregions of the NAc. The remaining 7 rats showed DREADDs expression exclusively in the NAc shell, and 4 of these rats were Drd1a-iCre while 3 were Drd2-iCre. No apparent behavioral differences were observed in these 7 rats and, as such, they were included with the rats expressing DREADDs in both the NAc core and shell. All animal procedures were carried out in accordance with the National Institutes of Health Guide for the Care and Use of Laboratory Animals and were approved by the Florida State University Institutional Animal Care and Use Committee.

### Genotyping

Tissue samples from ear punch biopsies were collected from rat pups on postnatal day 21 during weaning. To extract DNA, ear punches were dissolved in 1 µL Proteinase K (20 mg/mL, Millipore) in 200 µL of tissue lysis buffer and digested overnight at 55 ºC. The following day, tissue was treated with RNase A (2 mg/mL, CalBiochem) and purified using phenol–chloroform (VWR). DNA underwent ethanol precipitation after 3 M sodium acetate and 100% ethanol was added and then was incubated overnight at 4 ºC. A pellet was obtained after centrifugation at 10,000 rpm and resuspended in Ultrapure water (Invitrogen). DNA concentration was determined via absorbance spectroscopy measured on a NanoDrop (Thermo Fisher). PCR was performed on the DNA samples by using One*Taq* Hot Start polymerase (New England Biolabs) according to the manufacturer’s protocol and amplified in either a GeneAmp PCR System 9700 or an Eppendorf Mastercycler thermocycler. Primer information was obtained from the RRRC genotyping protocol #767 for Drd1a-iCre rats as follows: Drd1a F 5′-CTCCTGATGGAACCCTACCA-3′ and iCre R12 5′-CACAGTCAGCAGGTTGGAGA-3′. Primers for Drd2-iCre rats were obtained from RRRC #768 genotyping protocol as follows: Drd2 F 5′-TCAGGGAACCCTCTTTGAGA-3′ and iCre R12 5′-CACAGTCAGCAGGTTGGAGA-3′. PCR samples were electrophoretically run on an ethidium bromide DNA gel and imaged using a Bio-Rad ChemiDoc imaging system. Transgene-positive samples displayed a band at 202 base pairs (bp) for Drd1a-iCre rats or at 161 bp for Drd2-iCre rats, while transgene-negative samples (Wt) did not yield any bands (Fig. S1).

### Drugs

Alcohol (Koptec) was prepared by diluting ethyl alcohol solution in deionized (DI) water to 20% (40 proof) v/v from a 200-proof solution. For stereotaxic surgeries, bupivicaine and meloxicam analgesics were dissolved in sterile saline to obtain doses of 2.5 mg/mL and 1 mg/mL, respectively. For intraperitoneal (i.p.) injections, clozapine-N-oxide (CNO; Tocris) was dissolved in 100% dimethyl sulfoxide (DMSO) and then diluted with sterile saline to a final concentration of 3 mg/mL CNO in 5% DMSO solution. Vehicle solution consisted of 5% DMSO in sterile saline.

### Locomotor response to novelty

All rats underwent an initial 1-h novelty-induced locomotor test before experimental testing as previously described^46^. During the first 4-h of the dark cycle, rats were placed in circular chambers 71.2 cm in diameter (Med Associates) with four equidistant photobeam sensors that record locomotor movements based off number of beam breaks. This test allows categorization of rats into high- or low-responders to novelty based on locomotor scores above or below the median score for the cohort. This test was not used as an independent variable in any of the analyses but was taken into consideration when assigning and balancing experimental groups given that rats with differential responses to novelty were equally distributed between groups.

### Alcohol intake

The intermittent access to 20% alcohol 2-bottle choice (IA2BC20%) paradigm was used in the current study to model binge-like alcohol drinking in rodents as previously described with minor modifications^47^. Briefly, rats were given 24-h access to one bottle of 20% alcohol and one bottle of water. Drinking sessions started at the onset of the dark cycle (11 am) and occurred 3 days per week (Monday, Wednesday, Friday) for 8 weeks (see experimental timeline, Fig. 1a). The side placement of the alcohol bottle (left or right) was alternated each session to avoid the development of a side preference. On alcohol-deprivation days (Tuesday, Thursday, Saturday, Sunday), rats were exposed to two bottles of water. Prior to each drinking session, rats were weighed. To calculate the dose of alcohol consumed (g/kg) and the percent preference for alcohol (%), bottles were weighed before and after each session. The following equation was used to calculate g/kg of alcohol consumed (amount of alcohol consumed (mL) × 0.2)/(Body weight (kg)) at each time point measured. Percent preference was calculated using the following equation: (amount of alcohol consumed  (mL))/(total fluid consumed (mL) × 100).

Rats consumed alcohol for 4-weeks prior to intracranial delivery of either the stimulatory (hM3D(G_q_)) or inhibitory (hM4D(G_i_)) DREADDs viral constructs into the NAc. After a 3-day recovery period from surgery, rats re-initiated drinking under the IA2BC20% alcohol-drinking paradigm for 3-weeks to allow for optimal expression of the DREADDs constructs, which were serotype 8 AAV constructs driven by the human synapsin promoter for neuron-specific targeting^48^. During week-7 of alcohol intake, all rats regardless of viral construct or drug group were injected with DMSO 30-min before the start of the drinking session. This occurred during each session in week 7 (sessions 19–21) to allow the rats to adapt to injection stress and to provide a baseline by which rats drink alcohol after DMSO administration. During the first session of week 8 (session 22), rats were administered CNO or DMSO 30-min prior to the start of the drinking session. Bottles were weighed 1-, 4-, and 24-h after the introduction of the bottles to measure the effect that selective manipulation of NAc MSNs had on alcohol intake in Drd1a-iCre, Drd2-iCre, or wildtype (Wt) rats. Forty-eight hours after session 22, rats were administered a second injection of either CNO or DMSO, allowed access to alcohol and water bottles 30-min post-injection, and were terminated immediately after 60-min of alcohol intake (and 90-min after CNO or DMSO administration).

### Intracranial delivery of DREADDs

Twenty-four hours after the final drinking session of week 4 (session 12), rats were anesthetized with isoflurane gas (Henry Schein) at a concentration of 4.5% for induction and 3–4% for maintenance at an oxygen flow rate of 1 L/min. Rats were prepared for stereotaxic surgery under standard sterile conditions, and the skull was leveled on the dorsal–ventral plane by aligning bregma and lambda landmarks. DREADDs viral vectors were delivered into the NAc bilaterally at the following coordinates: A/P + 1.5 (from bregma), M/L ± 1.2, D/V − 7.6 (from the skull surface) as previously described^49,50^. We bilaterally injected 0.8 µL (1 × 10^12^ viral genomes/mL) of the Cre-recombinase-dependent viral vector carrying either the stimulatory (hM3D(G_q_)) or inhibitory (hM4D(G_i_)) designer receptors exclusively activated by designer drugs (DREADDs) into the nucleus accumbens (NAc) over 3.5 min at a rate of 0.1 µL every 30 s^51,52^. The DREADDs viral vectors were adeno-associated viruses that were serotype 8 and driven under the control of the human synapsin promoter to ensure neuron-specificity^49,53^. Additionally, vectors were Cre-dependent and were fused with the red fluorescent protein reporter, mCherry (Fig. 1b). The hM3D DREADD (pAAV8-hSyn-DIO-hM3D(G_q_)-mCherry, Addgene #44361-AAV8) is G_q_-coupled, inducing stimulatory effects when activated while the hM4D DREADD is G_i_-coupled (AAV8-hSyn-DIO-hM4D(G_i_)-mCherry, Addgene # 44362), inducing inhibitory effects when activated. After injecting viral constructs, the needle was left in place for an additional 5 min in each hemisphere to allow for sufficient diffusion. The needles were raised, craniotomies were sealed with bone wax, and the incision was sutured closed. Bupivicaine topical analgesic and triple antibiotic gel were applied to the incision site to assist with healing. Rats were administered meloxicam analgesic (5 mg/kg, i.p.) at the end of surgery. Rats underwent a 3-day recovery period before resuming the IA2BC alcohol-drinking paradigm for three more weeks.

### Tissue collection

Ninety minutes after administration of CNO or DMSO and 60-min after the introduction of alcohol and water bottles, rats were anesthetized with sodium pentobarbital (Henry Schein) diluted 1:1 in sterile saline via a single i.p. injection based on body weight. Rats were transcardially perfused with ice-cold 0.2 M phosphate buffered saline (PBS) followed by 4% paraformaldehyde (PFA) in PBS. Brains were extracted and post-fixed in 4% PFA for 24-h at 4 ºC before being transferred to a separate tube containing 0.2 M PBS + 0.1% sodium azide for long-term storage at 4 ºC. Serial coronal brain slices were collected at 40 µm thickness using a Leica vibratome (VT1200S). To confirm correct needle placement within the NAc, sections were temporarily placed onto a slide and viewed under a Leica DM6000 microscope with an XCite 120 PC fluorescent lamp using an N2.1 (red) filter cube at 10× objective. Brain sections containing mCherry reporter expression in the NAc were later used for RNAscope or immunohistochemistry assays, and those animals with mCherry expression outside of the NAc were removed from the study.

### RNAScope in situ hybridization assay

Fluorescent in situ hybridization (FISH) was performed to validate that iCre expression was localized to D1-MSNs in Drd1a-iCre rats and to D2-MSNs in Drd2-iCre rats. To do this, FISH was used to probe for the dopamine 1 receptor (Drd1a), the dopamine 2 receptor (Drd2), and mCherry, the red fluorescent protein fused to the Cre-dependent DREADDs constructs, mRNA according to the RNAScope Multiplex Fluorescent Reagent Kit v2 (Advanced Cell Diagnostics, #323100) user manual with minor modifications. Sections sliced at 40 µm containing mCherry-expressing neurons within the NAc were used and pretreated according to the formalin-fixed paraffin-embedded (FFPE) preparation method. We followed the pretreatment protocol for the tissue according to the manufacturer’s protocol, however because our tissue was not paraffin-embedded we skipped the deparaffinizing step and proceeded directly to the hydrogen peroxide step after baking the slides for 1-h. Tissue was treated for 30 min at 40 ºC with protease plus and washed it off with deionized (DI) water prior to adding the target probes for the Drd1a, Drd2, and mCherry RNA. Each probe contains a combination of 20 ZZ oligonucleotide probes bound to the target RNA (Rn-Drd1a-C1 (#317031) probe: GenBank Accession Number NM012546.2, target region 104-1053; Rn-Drd2-C2 (#315641) probe: GenBank Accession Number NM_012547.1, target region 455–1531; mCherry-C3 (#431201) probe: target region 23-681). After hybridization of the probes, pre-amplification and amplification probes were applied as follows: AMP1 30 min at 40  ºC, AMP2 30 min at 40 ºC, and AMP3 15 min at 40 ºC. Fluorescent dyes were then arbitrarily applied to specific channels. In the current study, tissue sections were probed with Drd1a and mCherry probes or Drd2 and mCherry probes. In Drd1a-iCre rats D1-MSNs were labeled with the Drd1a-C1 probe and assigned TSA Plus Cyanine 5 (Cy5, pink, Perkin Elmer #NEL745E001KT) to channel 1 while the cre-dependent DREADDs virus was labeled with the mCherry-C3 probe and the TSA Plus Cyanine 3 (Cy3, red, Perkin Elmer #NEL744E001KT) dye was assigned to channel 3 (Fig. 2a, Fig. S2). In Drd2-iCre rats, D2R-containing MSNs were labeled with the Drd2-C2 probe and assigned Cy5 to channel 2 while the mCherry-C3 probe along with Cy3 were assigned to channel 3 (Figs. 2b, S3). Sections were labeled with DAPI for 30-s before application of ProLong Gold mounting medium (ThermoFisher, #P36930) and cover-slipping with VWR micro cover glass (24 × 50 mm, No. 1.5). Tissue was washed in  1× wash buffer twice for 2-min each time in between incubations after probe hybridization steps. Prior to probe hybridization, tissue was washed with DI water as per the manufacturer’s instructions.

### Immunohistochemistry (IHC)

To validate DREADDs viral constructs, cFos protein expression was examined within mCherry-expressing DREADDs neurons to verify neuronal activation in hM3Dq-expressing neurons and neuronal silencing in hM4Di-expressing neurons. Given that rats were allowed access to alcohol 1-h prior to termination, we hypothesized that alcohol would increase cFos expression within the NAc and that activation of the hM4Di DREADD would reduce this while activating the hM3Dq DREADD would further increase cFos expression. To do this, double immunohistochemistry (IHC) was used to probe for cFos and mCherry. Tissue sections were sliced at 40 µm and slices containing DREADDs-mCherry expression within the NAc were used. Sections were initially washed 3 times with PBS, followed by a 1-h incubation period in 0.4% Triton X-100 in PBS with gentle agitation. Tissue was blocked for 1-h in a PBS solution containing 5% normal goat serum (NGS), 5% bovine serum solution (BSA), and 0.4% Triton X-100. Tissue was placed on a rocker overnight at 4 ºC in primary antibody (anti-mCherry, mouse, monoclonal, 1:750, Abcam #ab125096; anti-cFos, rabbit, monoclonal, 1:500, Cell Signaling #2250) in the blocking buffer mentioned above. The following day, the tissue was removed from the primary antibodies and washed in PBS + 0.4% Triton X-100 three times before being placed in secondary antibodies (Alexa Fluor Plus 488, donkey anti-rabbit, 1:500, ThermoFisher #A32790; Alexa Fluor 594, goat anti-mouse, 1:1000, ThermoFisher #A11005) overnight on a rocker at 4 ºC. Brain sections were labeled with DAPI for 30-s before being slide-mounted. ProLong Gold mounting medium was applied and the slides were cover-slipped with VWR micro cover glass (24 × 50 mm, No. 1.5).

### Image acquisition and quantification

For representative images collected to show target placement of the DREADDs-mCherry viral constructs within the NAc in Fig. 1b, slides were imaged at 2× and 4× objectives using a BZ-X710 Keyence fluorescent microscope. For FISH, images were acquired using a Zeiss LSM780 laser-scanning confocal using a 63× objective with a 1.4 oil immersion numerical aperture. Sections from Drd1a-iCre and Drd2-iCre rat brains were imaged to assess colocalization of D1-MSNs with the cre-dependent DREADDs fluorescent reporter, mCherry, for the Drd1a-iCre rat and D2-MSNs with mCherry in a Drd2-iCre rat. The Drd1a and Drd2 RNA were labeled with Cy5 dye and mCherry was labeled with Cy3 dye. DAPI staining was used to identify nuclei. Sixteen-bit z-stacks were collected at 2X Nyquist with a 0.2 µm z-step during excitation with the 405-laser (DAPI), the 528-laser (Cy3), and the 643-laser (Cy5). Z-stacks were loaded into Zen Blue and converted into TIFF files. Representative 2D snapshots that show co-labeling of the Cre-dependent DREADDs construct on D1-MSNs in a Drd1a-iCre rat and Cre on D2-MSNs in Drd2-iCre rats are shown in Fig. 2a,b. Given that some mCherry-expressing MSNs in Fig. 2a,b 2D snapshots do not have Drd1a or Drd2 mRNA co-labeling, maximum intensity projections and representative images through the 3D z-stack are shown in Figures S2 and S3 to show mRNA expression through the depth of the whole cell body. To acquire images for IHC, a Zeiss LSM780 laser-scanning confocal microscope was used at 20× objective. Sixteen-bit snapshots were collected during excitation with the 405-laser (DAPI), the 488-laser (cFos), and the 594-laser (mCherry). For IHC, at least four tissue sections containing NAc were imaged bilaterally (> 4 images per section in different regions of NAc) and within each section, total mCherry cells imaged per brain ranged from 100 to 400. Images were converted to TIFF files and loaded into Fiji for quantification. Within each image, the number of mCherry-expressing neurons and the total number of cFos-positive nuclei within these neurons were quantified.

### Statistical analyses

Behavioral and IHC data were analyzed with either GraphPad Prism (version 8) or RStudio (version 3.5.1). Data analysis in RStudio was performed using a linear mixed-model framework for ANOVAs with the packages nlme^54^ and lsmeans^55^. Behavioral measures analyzed prior to intra-NAc delivery of the DREADDs viral constructs such as body weight, total fluid intake, and food intake were analyzed using three-way linear mixed-model (LMM) ANOVA where sex and genotype were the between-subjects factor and session was the within-subjects factor. Analyses performed after DREADDs delivery used four-way LMM where sex, genotype, and virus were the between-subjects factor and session was the within-subjects factor. Alcohol intake and preference measured over the 8-week study as well as percent change in alcohol intake following DREADDs activation were analyzed using four-way LMM ANOVA. For LMM ANOVAs, time was treated as a linear vector to analyze the effect of sessions using a repeated measures mixed-model framework. Statistical outputs were considered significant when p < 0.05. Statistically significant main effects or interactions were followed up with Tukey’s post hoc tests for data analyzed in R and Sidak’s post hoc test for data analyzed in GraphPad.

Body weight gain was analyzed as a function of genotype using one-way ANOVA in GraphPad. IHC data was analyzed in GraphPad using two-way ANOVA where treatment and genotype were the between-subjects factors. Alcohol intake and total fluid intake during week 8 were analyzed using repeated measures (RM) two-way ANOVA fitted to a mixed model comparing sex and treatment in GraphPad. To assess changes in alcohol intake within the same rats after CNO administration compared to DMSO administration and to also assess sex differences in these effects, sex was treated as the between-subjects factor and treatment was the within-subjects factor. Analysis on these data were done separately for each time point alcohol intake was measured at (1-,4-, and 24-h). Average intake after DMSO administration in week-7 was obtained by averaging all three sessions if they were within 20% variability. If one session did not meet these criteria, the sessions within 20% variability were used to calculate an average. Statistical significance was set at α = 0.05 and all data is represented as mean ± SEM.

## Supplementary information

Supplementary Information.

Supplementary Figure S1.

Supplementary Figure S2.

Supplementary Figure S3.

Supplementary Figure S4.
