# Supplementary material for: Chemogenetic selective manipulation of nucleus accumbens medium spiny neurons bidirectionally controls alcohol intake in male and female rats
Source: Sci Rep. 2020 Nov 5;10:19178. doi: 10.1038/s41598-020-76183-2 (PMC7644642; doi:10.1038/s41598-020-76183-2)
Supplement: Supplementary file 1 — Supplementary Information. [file 41598_2020_76183_MOESM1_ESM.pdf]

Supplemental Results

Body weight, total fluid intake, food intake

Body weights, total fluid intake, and food intake were analyzed using three-way linear mixed model (LMM) ANOVAs comparing sex, genotype, and sessions while body weight gain from week 1-8 was analyzed using one-way ANOVA to compare between-genotypes within each sex. For body weight, total fluid, and food intake analyses, significant sex x sessions interactions revealed that male rats weighed more and consumed more total fluid and food on every session during experimental testing (body weight: $F_{(21,3337)} = 464.93$, $p < 0.0001$; total fluid: $F_{(21,3380)} = 3.53$, $p < 0.0001$; food: $F_{(21,3090)} = 2.26$; Tukey's post-hoc sessions 1-22 $p < 0.0001$). Each measure was separately analyzed within sex following significant sex x sessions interactions.

Within rats of both sexes, significant main effects of sessions were observed, indicating both male and female rats significantly increase body weights over the 8-weeks of testing (Males: $F_{(21,1741)} = 1639.45$, $p < 0.0001$; Females: $F_{(21,1596)} = 795.43$, $p < 0.0001$). In male rats, a main effect of genotype revealed that Drd1a-iCre rats weighed less overall compared to Drd2-iCre and Wt rats ($F_{(2,83)} = 10.05$, $p = 0.0001$, Tukey's post-hoc, Drd1a-iCre vs Wt: $t_{(83)} = -3.47$, $p = 0.002$, Drd1a-iCre vs Drd2-iCre: $t_{(83)} = -4.12$, $p = 0.0003$). In both sexes, body weight gain from weeks 1-8 was not impacted by genotype (Males: $F_{(2,82)} = 1.1$, $p = 0.34$; Females: $F_{(2,76)} = 0.75$, $p = 0.48$).

In male rats, a significant genotype x session interaction was followed up with Tukey's post-hoc to show that Drd1a-iCre males consume significantly less total fluid than Drd2-iCre and/or Wt on three sessions during the 8-weeks of alcohol intake ($F_{(42,1708)} = 1.66$, $p = 0.005$; session 3, Drd1a-iCre vs Wt: $t_{(84)} = -2.4$, $p = 0.047$; sessions 3,4,16, Drd1a-iCre vs Drd2-iCre: $t_{(84)} = -3.38$, -2.67, -2.52, $p = 0.003$, 0.024, 0.036). In female rats, a significant genotype x session interaction was followed up with Tukey's post-hoc to show that Drd2-iCre females consume significantly

more total fluid compared to Drd1a-iCre or Wt females on three sessions ($F_{(42,1672)}$ = 1.55, p = 0.014; session 3, Drd1a-iCre vs Drd2-iCre: $t_{(81)}$ = -2.46, p = 0.042; sessions 19, 20 Drd2-iCre vs Wt: $t_{(81)}$ = 2.41, 2.58, p = 0.047, 0.03).

A significant genotype x session interaction was followed up with Tukey's post-hoc to show that Drd1a-iCre male rats consumed less food than Drd2-iCre and/or Wt males throughout the 8-week period ($F_{(42,1582)}$ = 1.51, p = 0.02; sessions 1, 5, 7, 15, 16 , Drd1a-iCre vs Wt: $t_{(80)}$ = -2.64, -3.21, -2.82, -2.47, -2.55, p = 0.027, 0.005, 0.017, 0.04, 0.03; sessions 3, 5, 20, Drd1a-iCre vs Drd2-iCre: $t_{(80)}$ = -2.99, -2.94, -3.35, p = 0.01, 0.012, 0.004). In female rats, food intake was unaltered by genotype or session ($F_{(42, 1508)}$ = 1.27, p = 0.12).

Supplemental Figure Legends

Figure S1. Genotyping. Representative image of DNA gel. In Drd1a-iCre rats (left), the presence of a band at 202 base pairs (bp) indicates a transgene-positive rat (Drd1a-iCre) whereas the absence of a band indicates a transgene-negative rat (Wt). In Drd2-iCre rats (right), the presence of a band at 161 bp indicates a transgene-positive rat (Drd2-iCre) whereas the absence of a band indicates a transgene-negative rat (Wt).

Figure S2. Representative z-stack showing co-labeling of neurons with Drd1a and mCherry mRNA. (a-f.) 2D images of the XY plane as well as orthogonal XZ (top) and YZ (right) views shown to indicate depth in the 11 µm z-stack acquired with 1 µm z-steps. Arrows indicate cells with co-labeled Drd1a mCherry mRNA. **(a.)** Maximum intensity projection image of a z-stack labeled with DAPI (blue), Drd1a (green), and mCherry (red) mRNA. Cumulative co-labeled neurons indicated by arrows **(b-f.)** Representative images of specific z-slices within the 11 µm z-stack showing co-labeled Drd1a and mCherry neurons on different z-planes. Depth within the z-stack is indicated by number of µm distance from the top of the z-stack.

Figure S3. Representative z-stack showing co-labeling of neurons with Drd1a and mCherry mRNA. (a-f.) 2D images of the XY plane as well as orthogonal XZ (top) and YZ (right) views shown to indicate depth in the 21 μm z-stack acquired with 1 μm z-steps. Arrows indicate cells with co-labeled Drd2 mCherry mRNA. **(a.)** Maximum intensity projection image of a z-stack labeled with DAPI (blue), Drd2 (green), and mCherry (red) mRNA. Cumulative co-labeled neurons indicated by arrows **(b-f.)** Representative images of specific z-slices within the 21 μm z-stack showing co-labeled Drd1a and mCherry neurons on different z-planes. Depth within the z-stack is indicated by number of μm distance from the top of the z-stack.

Figure S4. Body weights, weight gain, total fluid intake, and food intake during 8-weeks of experimental testing. (a-f.) Male rats display significantly higher body weights, total fluid intake, and food intake compared to females. **(a,b.)** Drd1a-iCre male rats weigh significantly less than Drd2-iCre and Wt males during 8-weeks of alcohol intake, while no differences in genotype were observed in females. **(a,b inset)** Weight gain is similar between genotypes in rats of both sexes. **(c,d.)** In male rats, Drd2-iCre and Wt display significantly increased total fluid intake compared to Drd1a-iCre males on three sessions during the 8-week period. In females, Drd2-iCre rats display increased total fluid intake compared to Wt or Drd1a-iCre rats on three sessions throughout the 8-week period. **(e,f.)** In male rats, Drd2-iCre and Wt rats display increased food intake compared to Drd1a-iCre males on seven sessions. No differences in food intake were observed in females. Data represented as mean ± SEM. * Wt vs Drd1a-iCre $p < 0.05$, ^ Drd2iCre vs Drd1a-iCre $p < 0.05$, # Wt and Drd2-iCre vs Drd1a-iCre $p < 0.05$, @ Drd2iCre vs Wt $p < 0.05$.
