# Supplementary figures and images for: Chemogenetic selective manipulation of nucleus accumbens medium spiny neurons bidirectionally controls alcohol intake in male and female rats
Source: Sci Rep. 2020 Nov 5;10:19178. doi: 10.1038/s41598-020-76183-2 (PMC7644642; doi:10.1038/s41598-020-76183-2)

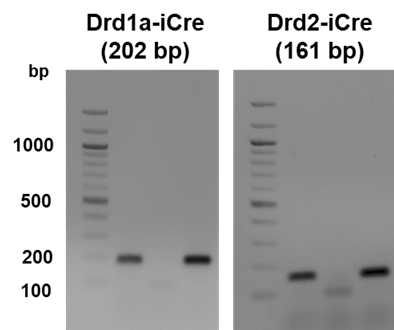

Supplement: Supplementary file 2 — Supplementary Figure S1. [file 41598_2020_76183_MOESM2_ESM.tif]

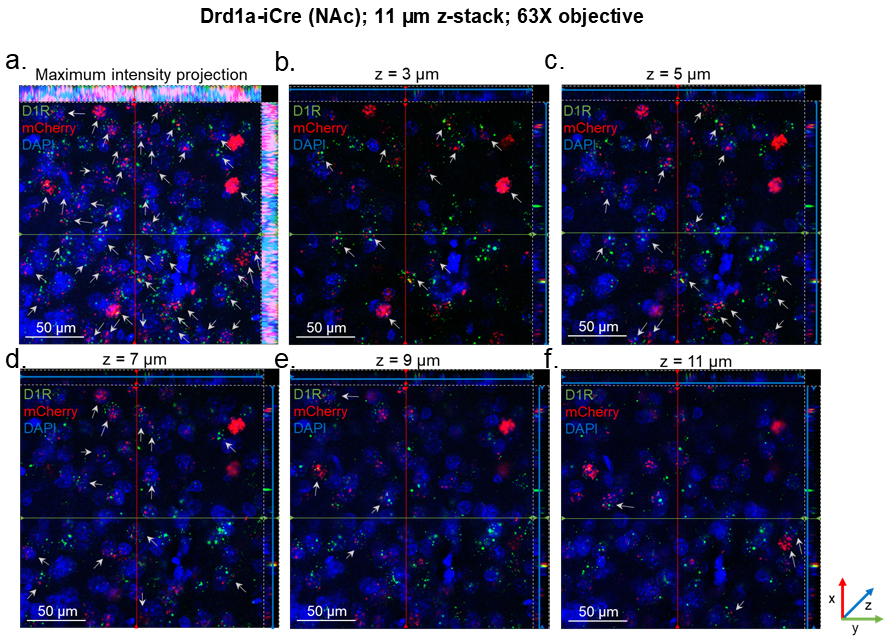

Supplement: Supplementary file 3 — Supplementary Figure S2. [file 41598_2020_76183_MOESM3_ESM.tif]

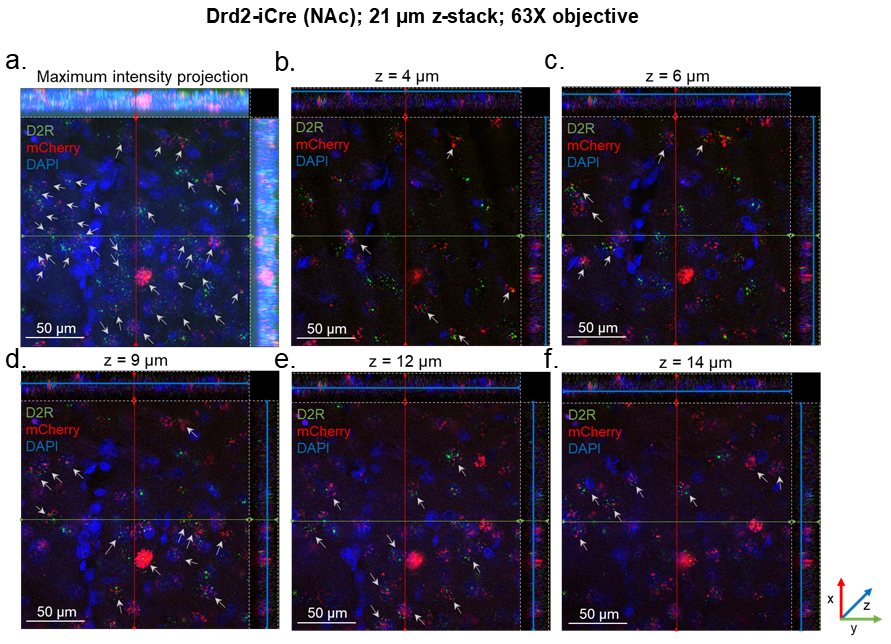

Supplement: Supplementary file 4 — Supplementary Figure S3. [file 41598_2020_76183_MOESM4_ESM.tif]

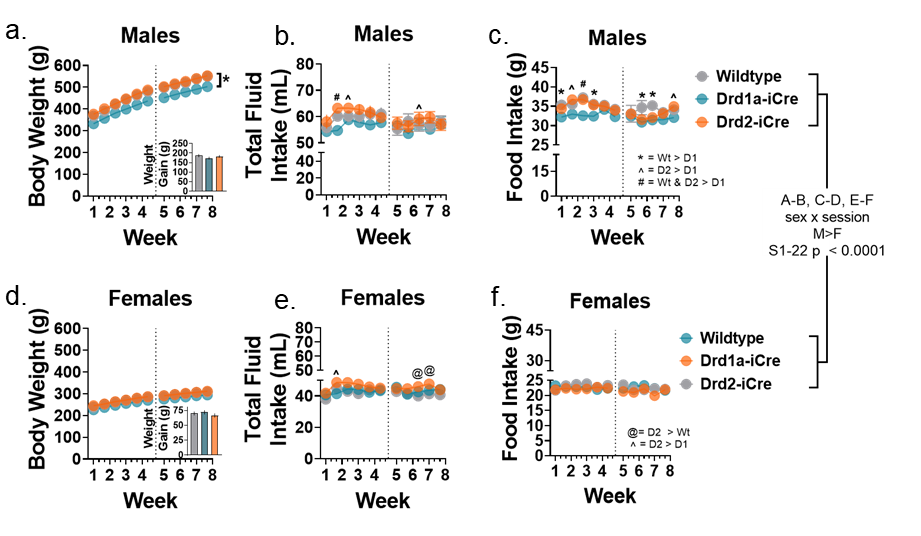

Supplement: Supplementary file 5 — Supplementary Figure S4. [file 41598_2020_76183_MOESM5_ESM.tif]
